# Phosphoinositol 3-kinase-driven NET formation involves different isoforms and signaling partners depending on the stimulus

**DOI:** 10.3389/fimmu.2023.1042686

**Published:** 2023-01-24

**Authors:** Vanessa de Carvalho Oliveira, Olga Tatsiy, Patrick P. McDonald

**Affiliations:** ^1^ Pulmonary Division, Faculty of Medicine, Université de Sherbrook and Centre de recherche du CHUS (CRCHUS), Sherbrooke, QC, Canada; ^2^ Department of Immunology and Cell Biology, Faculty of Medicine, Université de Sherbrooke and Centre de recherche du CHUS (CRCHUS), Sherbrooke, QC, Canada

**Keywords:** neutrophil extracellular traps, PI 3-kinase, PDK1, mTOR, PLCγ2, Akt

## Abstract

Neutrophil extracellular traps (NETs) serve to immobilize and kill pathogens, but also can contribute to the progression of several inflammatory and auto-immune diseases, as well as cancer. Whence the importance of elucidating the mechanisms underlying NET formation. In this regard, the PI3K signaling pathway has been shown to be crucial; yet little is known about which of its components are involved. Here, we identified the PI3K isoforms and associated signaling partners that are mobilized in response to different classes of physiological NET inducers (inflammatory cytokines, growth factors, chemoattractants). NET generation was assessed by microscopy and signalling molecule activation by immunoblot using phospho-antibodies. Across the various stimuli, PI3Kα and PI3Kγ isoforms clearly contributed to NET induction, while the participation of other isoforms was stimulus-dependent. Some PI3K isoforms were also found to signal through Akt, the canonical downstream effector of PI3K, while others did not. Downstream of PI3K, mTOR and PLCγ2 were used by all stimuli to control NET generation. Conversely, the involvement of other kinases depended on the stimulus – both TNFα and GM-CSF relied on PDK1 and Akt; and both TNFα and fMLP additionally used S6K. We further established that all PI3K isoforms and downstream effectors act belatedly in NET generation, as reported previously for PI3K. Finally, we revisited the PI3K-PDK1-Akt signaling hierarchy in human neutrophils and again found stimulus-dependent differences. Our data uncover unsuspected complexity and redundancy in the signaling machinery controlling NET formation through the all-important PI3K pathway. Conserved signaling molecules represent therapeutic targets for pathologies involving NETs and in this regard, the existence of drugs currently used in the clinic or undergoing clinical trials (which target PI3K isoforms, mTOR or Akt), underscores the translational potential of our findings.

## Introduction

Peripheral blood neutrophils circulate in large numbers, surveilling for injury and pathogens. Intruders facing neutrophils can be engulfed and digested by phagocytosis, and/or encounter various antimicrobial peptides and enzymes originating from intracellular granules, as well as reactive oxygen species which attack most components of living organisms. In addition, viruses, bacteria, fungi, and parasites can be immobilized and inactivated by neutrophil extracellular traps (NETs) – extruded webs of decondensed chromatin adorned with numerous antimicrobial peptides and proteolytic enzymes (1, 2). The central role of NETs in host defense is well established (3, 4). Conversely, NETs also contribute to the progression of a vast range of autoimmune, inflammatory, and cardiovascular diseases, as well as cancer (5). More recently, NETs were shown to participate in the pathological progression of Covid-19 infections. For example, SARS-CoV-2-induced NETs trigger apoptosis of lung epithelial cells, which engenders acute lung injury (6). Thrombosis is another COVID-19 complication that is facilitated by NETs, which serve as a scaffold and mediator for thrombus formation (7–9). Since NETs exert such diverse actions, both in health and disease, exploring the molecular mechanisms underlying their formation is both timely and relevant.

A pivotal molecule driving NET formation is phosphoinositide 3-kinase (PI3K) (10–14). This holoenzyme remains active throughout most of the multi-hour process culminating in NET generation; in this regard, its inhibition at 2 h post-stimulation can still prevent NET release (13). Active PI3K phosphorylates PI(4, 5)P_2_, thereby creating membrane docking sites for proteins that possess appropriate adaptor domains, such as the pleckstrin homology (PH) domain. Some prominent examples include Akt which among other actions promotes cell survival; GEFs (guanine nucleotide exchange factors) which can associate with small G proteins such as Rho/Rac to control cytoskeletal rearrangements; and PDK1 which can phosphorylate key cellular kinases including Akt, ribosomal S6 kinase, and PKC isoforms (reviewed in (15)). Structurally, PI3K enzymes consist of regulatory and catalytic subunits. Class IA PI3Ks are heterodimers composed of a p110α, p110β or p110δ catalytic subunit, and of a regulatory subunit (often p85); the latter features SH2 domains that allow an association with receptor tyrosine kinases (RTKs), such as cytokine and growth factor receptors. By comparison, class IB PI3Ks feature a p110γ catalytic subunit and their regulatory subunit can be directly bound by the Gβγ moiety of activated G protein-coupled receptors (GPCRs). All PI3K isoforms are expressed by neutrophils (16). We and others have shown that in these cells, a class IA PI3K (p85α/p110δ or p85α/p110β) drives LPS- or TNF-induced responses such as inflammatory cytokine production, delayed apoptosis, adherence, NADPH oxidase activation, and degranulation (16–18). Conversely, class IB PI3Ks appear to mediate neutrophil responses triggered by GPCR ligands such as fMLF or CXCL8 (19, 20).

Despite the crucial role played by PI3K in NET induction by a variety of stimuli, little is known of how various PI3K isoforms or PH domain-containing signaling partners participate in the phenomenon. In this regard, it was reported that PI3Kδ mediates NET formation elicited by fungi or parasites (21, 22). Downstream of PI3K, parasite-induced NETs did not rely on Akt, while the latter appears to be essential for PMA-triggered NET formation (21, 23–25). As for mTOR, an intermediate downstream of Akt, there are conflicting reports about its role in NET production (23, 26, 27). While these scattered reports paint a fragmentary portrait of how PI3K system might control NET formation, this core issue still awaits a systematic investigation. In this study, we dissected the contribution of individual PI3K isoforms and associated signaling molecules to NET induction by discrete classes of physiological stimuli. We now report that there exist conserved NET signaling kinases (PI3Kα, PI3Kγ, PLCγ2, mTOR) driving NET formation and that conversely, other signaling molecules (PI3Kβ, PI3Kδ, PDK1, Akt, S6K) are differentially mobilized depending on the stimulus.

## Materials and methods

### Antibodies and reagents

Antibodies against P-Akt (#4060), P-mTORC1 (#5536), P-S6K (# 9234), P-PLCγ2 (#3871), and β-actin (#4967) were from Cell Signaling (Beverly, MA, USA); a pan histone antibody was from Sigma (#MABE71). Ficoll-Paque Plus was from GE Biosciences (Baie d’Urfé, Qc, Canada); Dextran T500 from Pharmacosmos (Holbæk, Denmark); endotoxin-free (< 2 pg/ml) RPMI 1640 from Wisent (St-Bruno, Qc, Canada); poly-L-lysine from Peptides International (Louisville, KY, USA). Recombinant human cytokines were from R&D Systems (Minneapolis, MN, USA. Dimethyl sulfoxide (DMSO), N-formyl-methionyl-phenylalanine (fMLF), and phenylmethanesulphonyl fluoride (PMSF) were from Sigma-Aldrich (St. Louis, MO, USA). All inhibitors were purchased through Cedarlane Labs (Missisauga, Canada). ProLong Gold Antifade, Hoechst 33342, propidium iodide, and 16% paraformaldehyde (PFA) were purchased from Thermo Fisher (Missisauga, Canada). PlaNET reagents (fluorescent chromatin-binding polymers) are no longer available from Immune Biosolutions or other suppliers; we therefore employed a close equivalent – fluorescent, 50-nm carboxylate microspheres (# 16661-10) from Polysciences Inc. (Warrington, PA, USA). These microspheres are referred to as PlaNET reagents throughout this study. All other reagents were of the highest available grade, and all buffers and solutions were prepared using pyrogen-free clinical grade water.

### Cell isolation and culture

Neutrophils were isolated from the peripheral blood of healthy donors, following a protocol that was duly approved by an institutional ethics committee. All subjects gave written informed consent in accordance with the Declaration of Helsinki. Briefly, whole blood was collected using an anticoagulant (sodium citrate), and successively submitted to dextran sedimentation, Ficoll separation, and water lysis – as described previously (28). The entire procedure was carried out at room temperature and under endotoxin-free conditions. Purified neutrophils were resuspended in RPMI 1640 supplemented with 10% autologous serum, at a final concentration of 5 x 106 cells/ml (unless otherwise stated). As determined by Wright staining and FACS analysis, the final neutrophil suspensions contained fewer than 0.2% monocytes or lymphocytes; neutrophil viability exceeded 98% after up to 4 h in culture, as determined by trypan blue exclusion and by Annexin V/propidium iodide FACS analysis.

### NET microscopic assays

For each condition, 500 µl of a neutrophil suspension (2x106/ml in RPMI 1640/2% autologous serum) was deposited onto coverslips that had been freshly coated with poly-L-lysine and placed inside the wells of a 24-well plate; the cells were then left to adhere for 60 min in a cell culture incubator. Cells were gently washed with pre-warmed culture medium and covered with 500 µl of fresh, pre-warmed medium. Inhibitors and/or stimuli were then added, and the final volume brought to 550 µl, prior to a 4-h incubation (37°C, 5% CO2). Reactions were stopped by adding 500 µl ice-cold PBS containing 1 mM PMSF, and the coverslips were placed on ice for 10 min. The liquid on the coverslips was discarded and cells were incubated (90 min on ice, with gentle shaking) in 1 ml of cold PBS containing 1 mM PMSF and 0.2 µl PlaNET reagent. Cells were finally fixed (15 min, room temperature) in PBS containing 2% parafornaldehyde, as well as a nuclear stain. The fixed cells were then washed with PBS, and the coverslips mounted onto glass slides using a drop of mounting medium (ProLong Gold, Life Technologies), prior to epifluorescence microscopy analysis. For quantitation, 3 fields at 10x magnification were typically counted, that never included the coverslip edges: this amounts to counting about 1,000 neutrophils per coverslip, or about 2000 cells per experimental condition since experiments are conducted in duplicate. Fluorescence quantitation was done using a Java plug-in for ImageJ which we developed (available at http://mcdonaldlab.ca/java-plug-in.html).

Unlike generic DNA dyes (e.g. Sytox Green, DAPI, Hoechst 33342), PlaNET reagents specifically bind extracellular, decondensed chromatin as we previously established (13). We moreover showed that detecting NETs using PlaNET Green or NET-specific CitH3 antibodies yields the same information (29). As shown in **Figure S1**, staining resting and fMLF-activated neutrophils with both PlaNET Green and a pan-histone Ab likewise results in NETs that are colored by both reagents. This further confirms that the PlaNET Green signal colocalizes with NET markers.

### Nuclear decondensation analyses

Chromatin decondensation was measured and quantitated exactly as described (29).

### Immunoblots

Neutrophils were incubated as described in the figure legends. Incubations were stopped by adding equivalent volumes of ice-cold PBS supplemented protease and phosphatase inhibitors and then placing on ice for at least 10 min, as previously described (30, 31). Cells were pelleted (2000 g, 5 min, 4°C), resuspended in boiling sample buffer, and incubated 5 min at 95°C. Samples were electrophoresed, transferred onto nitrocellulose, and processed for immunoblot analysis as previously described (30, 31). Samples thus prepared were sonicated to disrupt chromatin, and stored at –20°C prior to analysis.

### Data analysis

All data are represented as the mean ± SEM of at least three independent experiments. Unless otherwise stated, statistical differences were determined by Student’s t test for paired data; for such analyses, data distribution passed the Shapiro-Wilk normality test. All statistical analyses were performed using Prism 9 software (GraphPad Software, San Diego, CA, USA).

## Results

### PI3K isoforms differentially impact NET formation in a stimulus-dependent manner

**Figure 1:**
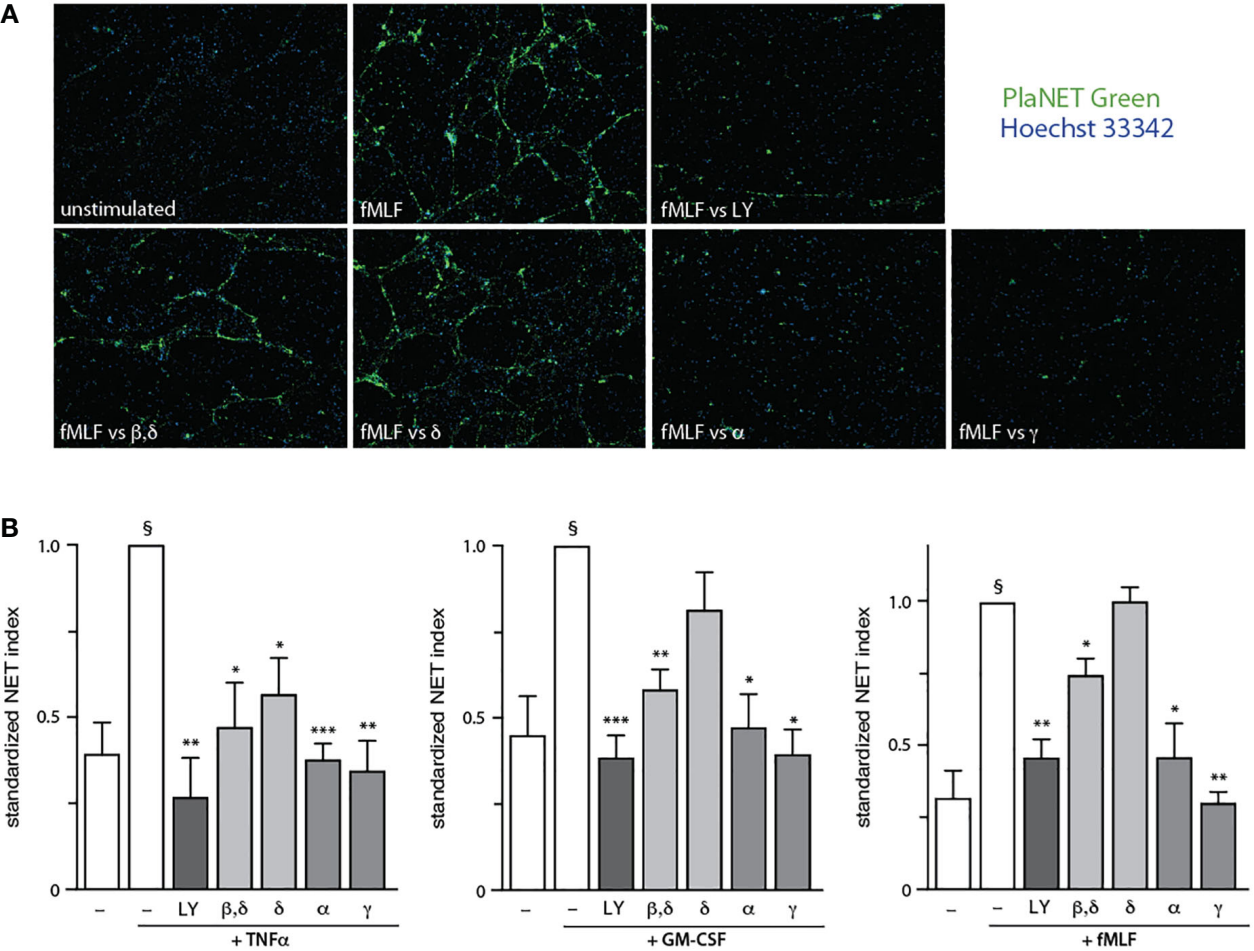
Stimulus-dependent involvement of discrete PI3K isoforms in NET formation. Neutrophils cultured at 37°C on poly-L-lysine-coated coverslips were pre-treated (15 min) with the following PI3K isoform-selective inhibitors or their diluent (0.1% DMSO): 10 μM LY294002 (a general PI3K inhibitor, “LY”); 1 μM TGX-221 (β, δ); 1 μM IC87114 (δ); 1 μM PI3Kα inhibitor IV (α); 1 μM AS64002 (γ). The cells were then stimulated or not for 4 h at 37°C with 100 U/ml TNFα, 1 nM GM-CSF, or 100 nM fMLF. NET formation was assessed using PlaNET Green as described in *Methods*. **(A)** A representative experiment is shown (10X magnification) for fMLF-treated cells. **(B)** Mean ± s.e.m. of the standardized NET index from at least 4 independent experiments. *, p<0.05; **, p<0.01; ***, p<0.001 vs stimulus alone. §, p < 0.02 for unstimulated cells vs stimulus.

**Figure 2:**
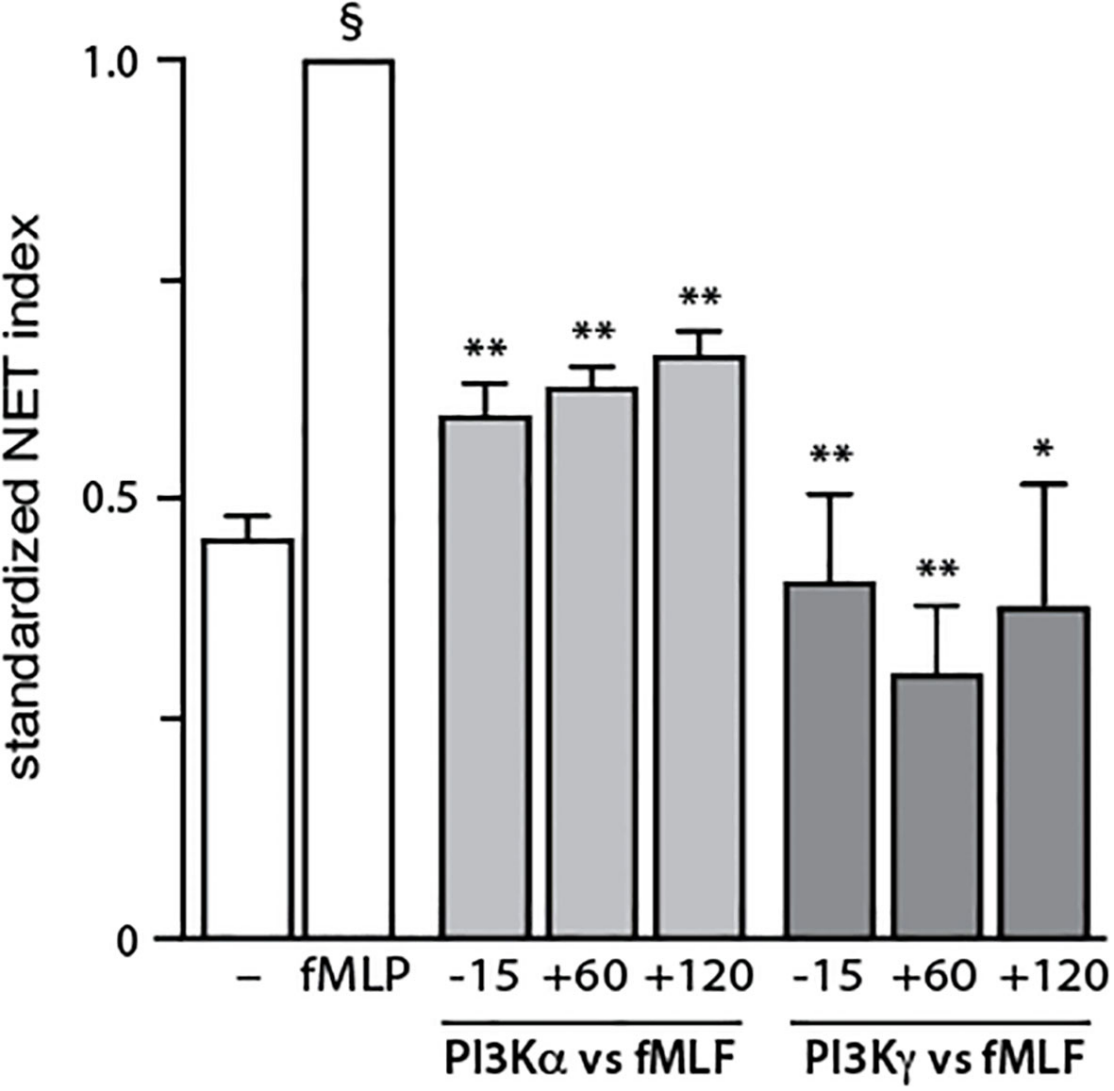
Belated inhibitory effect of PI3K isoforms on NET formation. Neutrophils cultured at 37°C on poly-L-lysine-coated coverslips were exposed to 1 μM PI3Kα inhibitor IV (PI3Kα-selective) or 1 μM AS64002 (PI3Kγ-selective), either before (-15 min) or into (+60 or +120 min) a 4-h stimulation with 100 nM fMLF. NET formation was assessed using PlaNET Green as described in *Methods*. Mean ± s.e.m. of the standardized NET index from 3 independent experiments. *, p<0.05; **, p<0.01 vs stimulus alone. §, p < 0.001 for unstimulated cells vs stimulus.

To identify the PI3K isoforms participating in NET formation, we relied on highly selective pharmacological inhibitors (listed in **Table S1**). We also employed different classes of physiological NET inducers – TNFα, a foremost inflammatory cytokine; GM-CSF, a prototypical growth factor; and fMLF, a classical chemoattractant. All stimuli could mobilize the various PI3K isoforms to cell membranes, albeit to varying degrees (**Figure S2**). As shown in **Figure 1**, PI3Kα and PI3Kγ isoforms clearly contribute to NET formation elicited by all three stimuli. But there were stimulus-dependent differences as well. For TNFα, PI3Kδ (and perhaps PI3Kβ) also mediate NET induction (**Figure 1B**, left panel) whereas for GM-CSF or fMLF, PI3Kβ was involved but PI3Kδ was not (**Figure 1B**, middle and rights panels). Because we previously established that PI3K contributes to NET formation by various stimuli as late as some 2 h into the process (13, 14), we investigated whether the PI3K isoform-selective inhibitors that prevent the phenomenon also act in a belated manner. For this purpose, the inhibitors were added either before or after stimulation; we focused on PI3Kα and PI3Kγ isoforms because they yielded the greatest inhibition and were mobilized by all stimuli tested. As depicted in **Figure 2**, the belated (+120 min) inhibition of the PI3Kα and PI3Kγ isoforms hinders fMLF-triggered NET formation similarly to when inhibitors are added at earlier times; differences between the times of inhibitor addition (-15 vs +60 vs +120 min) were indeed not found to be statistically significant by one-way ANOVA analysis with Tukey’s multiple comparisons test. A similar outcome was observed when TNFα or GM-CSF were used as NET inducers instead (**Figure S3**). This confirms the late action of PI3K towards NET production, regardless of the isoforms being mobilized. Since chromatin decondensation is a late event that precedes its extrusion (leading to NET formation), we examined whether PI3K isoform-selective inhibitors also affect decondensation. As shown in **Figure S4**, most inhibitors that affected NET formation also hindered chromatin decondensation, with the exception of TGX-221 in GM-CSF-treated cells. The PI3Kα inhibitor was also less potent in inhibiting decondensation in cells stimulated with GM-CSF or fMLF, than it was in blocking NET formation. In this regard, there exists evidence that chromatin decondensation and its subsequent extrusion can be regulated separately (32). Whether this is the case for the above inhibitors will be investigated in future studies (**Figure 8**).

### Discrete PI3K isoforms control downstream Akt phosphorylation, depending on the stimulus

**Figure 3:**
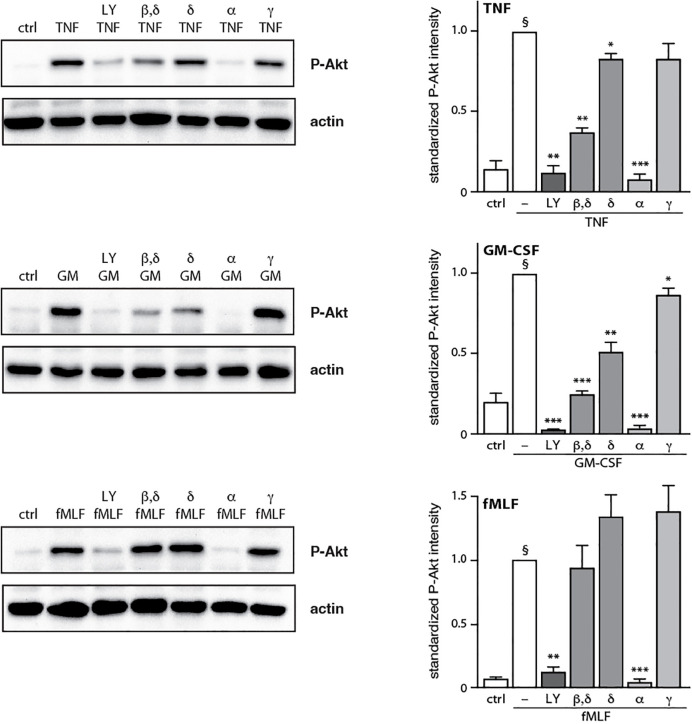
Effect of PI3K isoform inhibition on Akt phosphorylation. Neutrophils were pre-treated (15 min, 37°C) with the following PI3K isoform-selective inhibitors or their diluent (0.1% DMSO): 10 μM LY294002 (general PI3K inhibitor); 1 μM TGX-221 (β, δ); 1 μM IC87114 (δ); 1 μM PI3Kα inhibitor IV (α); 1 μM AS64002 (γ). The cells were then stimulated or not (“crtl”) for 10 min at 37°C with 100 U/ml TNFα, 1 nM GM-CSF, or 100 nM fMLF. Samples were processed for immunoblot analysis of P-Akt (S473) as well as β-actin (loading control). Representative blots are shown, along with compiled data (mean ± s.e.m.) from at least 3 independent experiments (in which P-Akt values were standardized versus actin and the resulting ratios were divided by that of the corresponding stimulus). *, p<0.05; **, p<0.01; ***, p<0.001 vs stimulus alone. §, p < 0.003 for unstimulated cells vs stimulus.

We next evaluated how the PI3K isoform-specific inhibitors affect the phosphorylation of the archetypal downstream effector of PI3K, Akt. Neutrophils were pre-treated with isoform-selective inhibitors, prior to stimulation and subsequent immunoblot analysis of P-Akt. As shown in **Figure 3**, PI3Kα blockade obliterated Akt phosphorylation regardless of the stimulus used (as did the general PI3K inhibitor, LY294002), whereas PI3Kγ inhibition failed to do so. Again, stimulus-dependent differences were observed. For TNFα, PI3Kβ also participates in Akt phosphorylation (**Figure 3**, top panel) whereas for GM-CSF, both PI3Kβ and PI3Kδ were also involved (**Figure 3**, middle panel). These data show that as in the case of NET formation, inducible Akt phosphorylation involves discrete PI3K isoforms depending on the stimulus used. However, the isoforms contributing to either response do not always match.

### Discrete PI3K signaling partners affect NET formation depending on the stimulus

**Figure 4:**
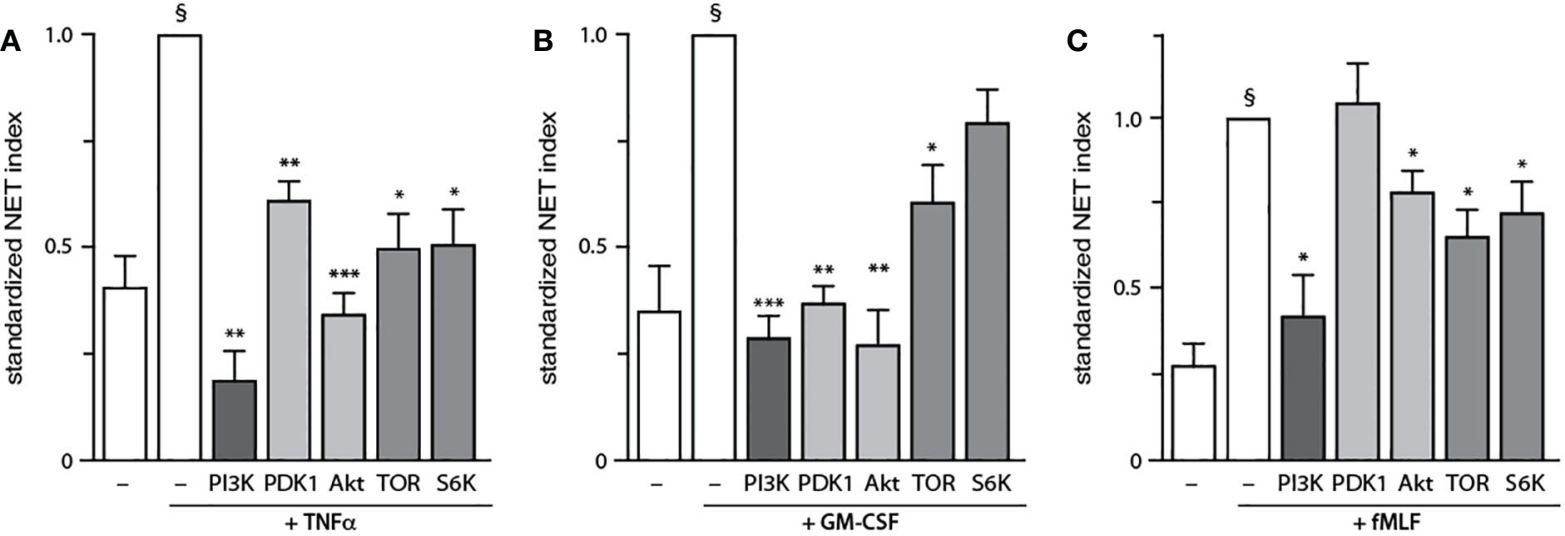
Discrete PI3K signaling partners drive NET formation depending on the stimulus. Neutrophils cultured on poly-L-lysine-coated coverslips were pre-treated (15 min, 37°C) with the following inhibitors or their diluent (0.1% DMSO): 10 μM LY294002 (PI3K inhibitor); 5 μM BX-912 (PDK1 inhibitor); 10 μM Akt inhibitor VIII (Akt inhibitor); 100 nM rapamycin (mTOR inhibitor); or 10 μM PF-4708671 (S6K inhibitor). The cells were then stimulated or not for 4 h at 37°C with **(A)** 100 U/ml TNFα, **(B)** 1 nM GM-CSF, or **(C)** 100 nM fMLF. NET formation was assessed using PlaNET Green as described in *Methods*. Mean ± s.e.m. from at least 3 independent experiments. *, p<0.05; **, p<0.01; ***, p<0.001 vs stimulus alone. §, p < 0.02 for unstimulated cells vs stimulus.

We next examined how NET formation is affected by individual signaling intermediates within the PI3K-Akt axis, using various pharmacological inhibitors (depicted in **Figure S5**). We initially conducted dose response experiments to determine the minimal inhibitor concentration needed to achieve a maximal effect in human neutrophils (**Figure S6**), as we had done done previously for the general PI3K inhibitor, LY-294002 (16). As shown in **Figure 4A**, inhibitors of PDK1, Akt, mTOR, and S6K all diminish TNF-elicited NET generation. A similar pattern was observed using GM-CSF, with the exception that S6K inhibition did not influence NET induction (**Figure 4B**). In fMLF-activated cells however, PDK1 was found to be uncoupled from NET formation while mTOR and S6K partially affected the phenomenon (**Figure 4C**). Because the PDK1 inhibitor used in **Figure 4** (BX-912) can also inhibit PKA at the concentration used (5 μM) (33), we employed two other PDK1-selective inhibitors (BX-795 and GSK 2334470) which do not inhibit PKA. As shown in **Figure S7**, this confirmed that PDK1 is required for NET production triggered by TNFα and GM-CSF, but not fMLF. Thus, PI3K-related signaling intermediates differentially control NET generation depending on the stimulus used.

**Figure 5:**
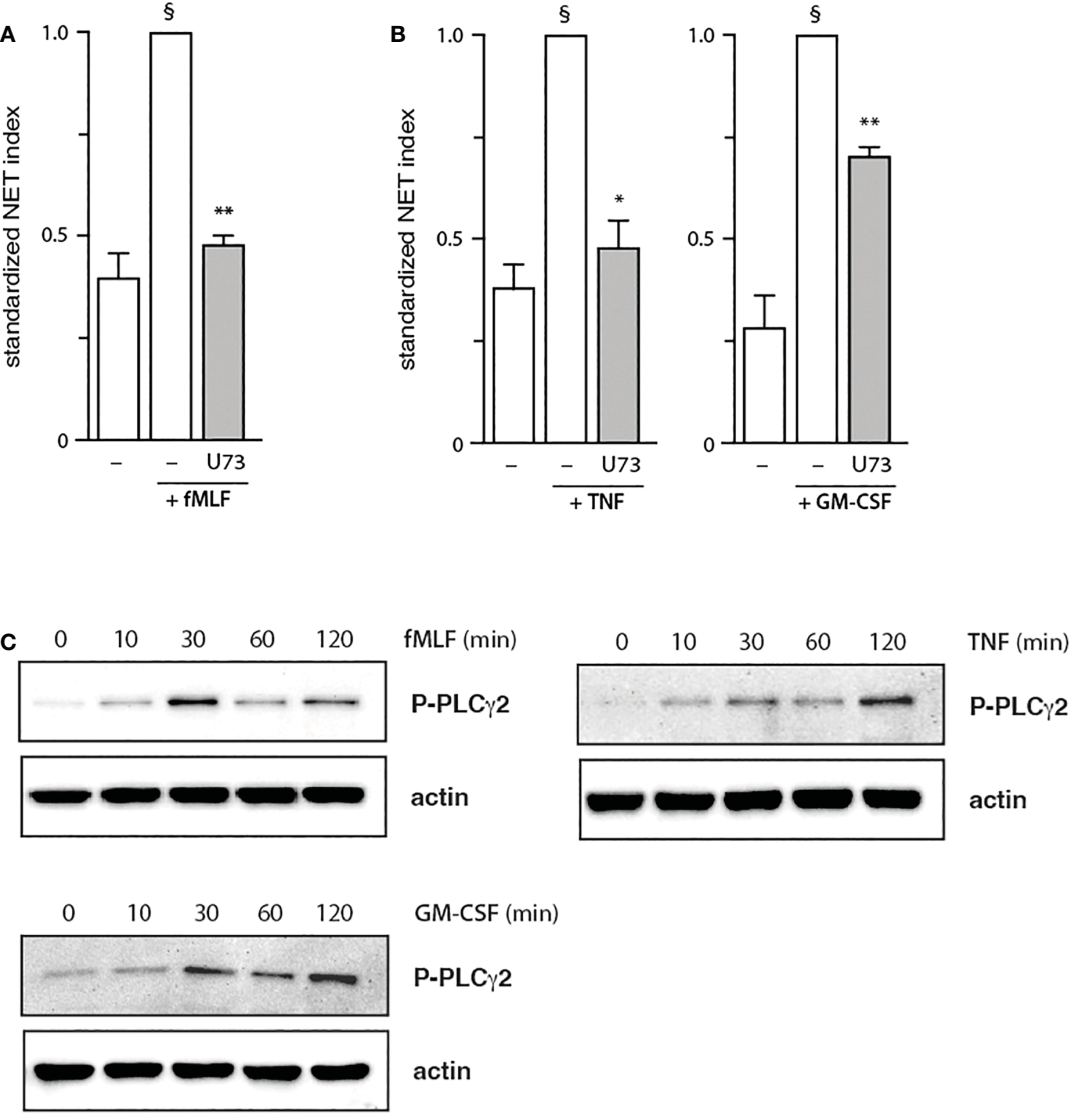
Role of PLCγ2 in NET formation. Neutrophils cultured on poly-L-lysine-coated coverslips were pre-treated (15 min, 37°C) with 1 μM U73122 (“U73”) or its diluent (0.1% DMF), prior to a 4-h stimulation with 100 nM fMLF **(A)** or with 100 U/ml TNFα, or 1 nM GM-CSF **(B)**. NET formation was assessed using PlaNET Green as described in *Methods*. Mean ± s.e.m. from at least 3 independent experiments. *, p<0.05; **, p<0.01 vs stimulus alone. §, p < 0.02 for unstimulated cells vs stimulus. **(C)** Adherent neutrophils were stimulated for the indicated times at 37°C with 100 nM fMLF, 100 U/ml TNFα, or 1 nM GM-CSF. Samples were processed for immunoblot analysis of P-PLCγ2 (Y1217) as well as β-actin (loading control). Representative blots are shown.

**Figure 6:**
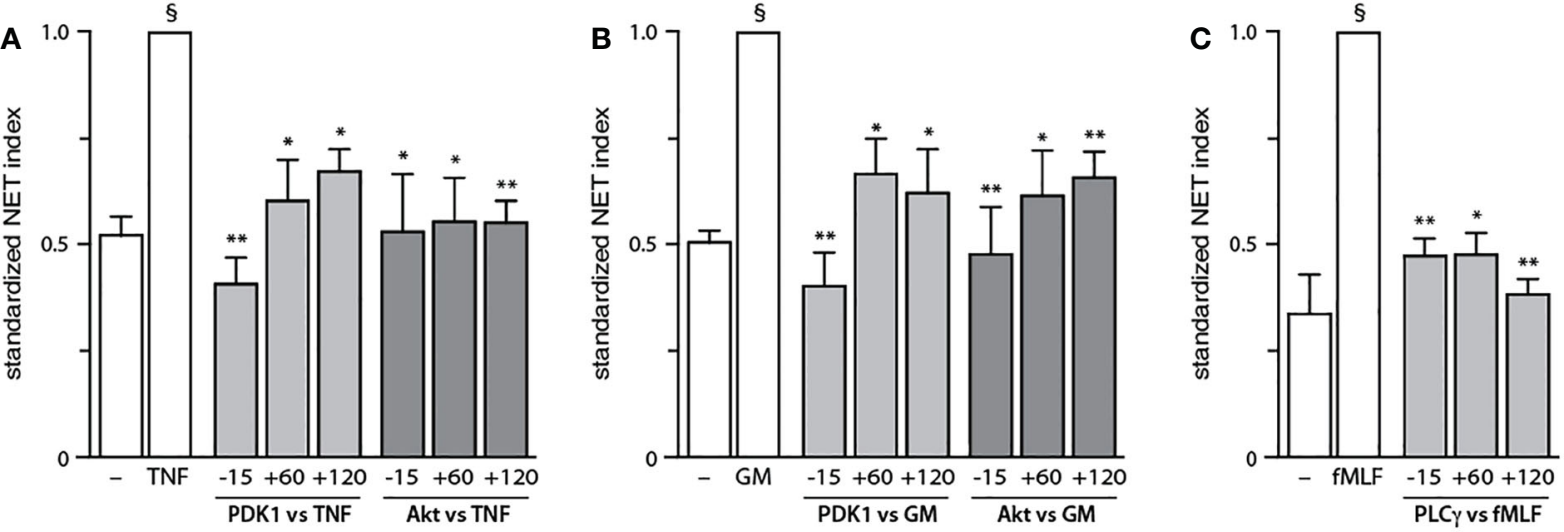
Belated contribution of PDK1, Akt, and PLCγ to NET generation. Neutrophils cultured at 37°C on poly-L-lysine-coated coverslips were treated either before (-15 min) or after (+60 or +120 min) stimulation with the following inhibitors or their diluent (0.1% DMSO): 5 μM BX-912 (PDK1 inhibitor); 10 μM Akt inhibitor VIII (Akt inhibitor); or 1 μM U73122 (PLCγ inhibitor). The cells were stimulated or not for 4 h at 37°C with **(A)** 100 U/ml TNFα, **(B)** 1 nM GM-CSF, or **(C)** 100 nM fMLF. NET formation was assessed using PlaNET Green as described in *Methods*. Mean ± s.e.m. from at least 3 independent experiments. *, p<0.05; **, p<0.01 vs stimulus alone. §, p < 0.003 for unstimulated cells vs stimulus.

The finding, that PDK1 does not mediate fMLF-triggered NET formation, prompted us to investigate other signaling partners that could be mobilized downstream of PI3K activation. Among those featuring a PH domain, PLCγ2 stood out as it is reportedly activated by fMLF in neutrophils (34), whereas cytokines and growth factors are not known to activate PLC in these cells. As shown in **Figure 5A**, PLCγ2 blockade efficiently blocked NET induction by fMLF; an optimal effect was observed using 1 µM of the inhibitor (**Figure S8**) despite the use of higher concentrations in the literature (10 µM of the inhibitor compromised cell viability in our hands). Unexpectedly, we found that PLCγ2 also participates in NET formation elicited by TNFα or GM-CSF (**Figure 5B**). We also confirmed that all three stimuli can induce PLCγ2 phosphorylation of (**Figure 5C**). Thus, all stimuli used herein signal through Akt and mTOR (to a variable degree), as well as PLCγ2, to elicit NET formation; TNFα and GM-CSF additionally rely on PDK1 for NET induction, while fMLF and TNFα also mobilize S6K to do so. Finally, we confirmed that the major PI3K signaling partners identified above affect NET formation in a belated manner (**Figure 6**), as does PI3K (13, 14). Differences between the times of inhibitor addition (-15 vs +60 vs +120 min) were indeed not found to be statistically significant by one-way ANOVA analysis with Tukey’s multiple comparisons test, except in the case of the PDK1 inhibitor vs GM-CSF, where addition at -15 min differed significantly (p<0.02) with inhibitor addition at +60 min, but not at +120 min. Consistent with this observation, both Akt and mTOR remain inducibly phosphorylated within this time frame (i.e. 90-120 min) in adherent neutrophils (like the ones that NET) exposed to all stimuli under study (**Figure S9**), as does PLCγ2 (**Figure 5B**). By comparison, PDK1 is constitutively phosphorylated and cell stimulation does not alter this state (not shown).

### The signaling hierarchy within the PI3K cascade depends on the stimulus in human neutrophils

**Figure 7:**
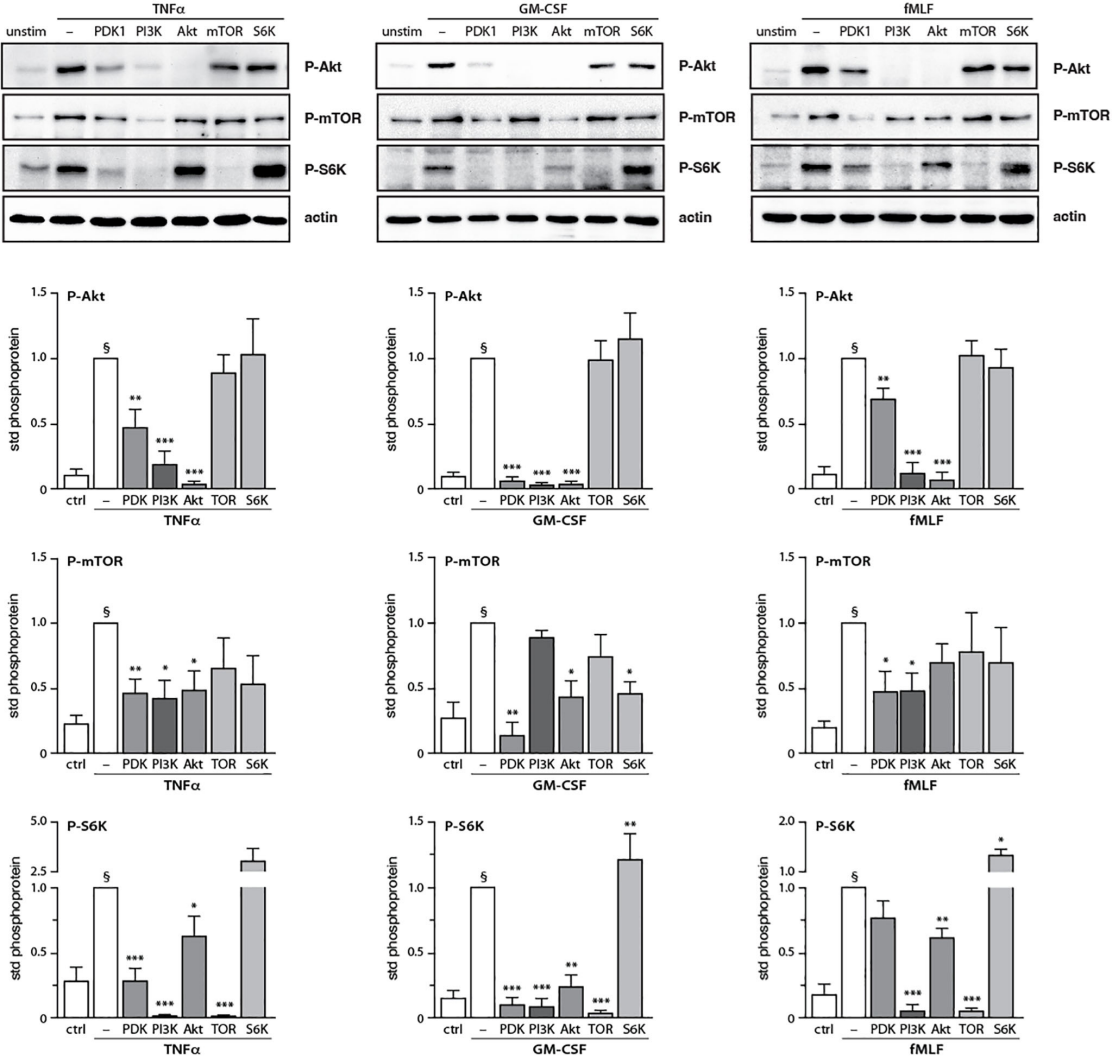
Inter-relationships between PI3K and its signaling partners in human neutrophils. Neutrophils were pre-treated (15 min, 37°C) with the following inhibitors or their diluent (0.1% DMSO): 10 μM LY294002 (PI3K inhibitor); 5 μM BX-912 (PDK1 inhibitor); 10 μM Akt inhibitor VIII (Akt inhibitor); 100 nM rapamycin (mTOR inhibitor); or 10 μM PF-4708671 (S6K inhibitor). The cells were then stimulated or not (10 min, 37°C) with 100 U/ml TNFα, 1 nM GM-CSF, or 100 nM fMLF. The samples were processed for immunoblot analysis of P-Akt (S473), P-mTOR (S2448), P-S6K (T389), as well as β-actin (loading control). Representative blots are shown, along with compiled data (mean ± s.e.m.) from at least 3 independent experiments (in which phosphoprotein values were standardized versus actin and the resulting ratios were divided by that of the corresponding stimulus). *, p<0.05; **, p<0.01; ***, p<0.001 vs stimulus alone. §, p < 0.02 for unstimulated cells vs stimulus.

**Figure 8:**
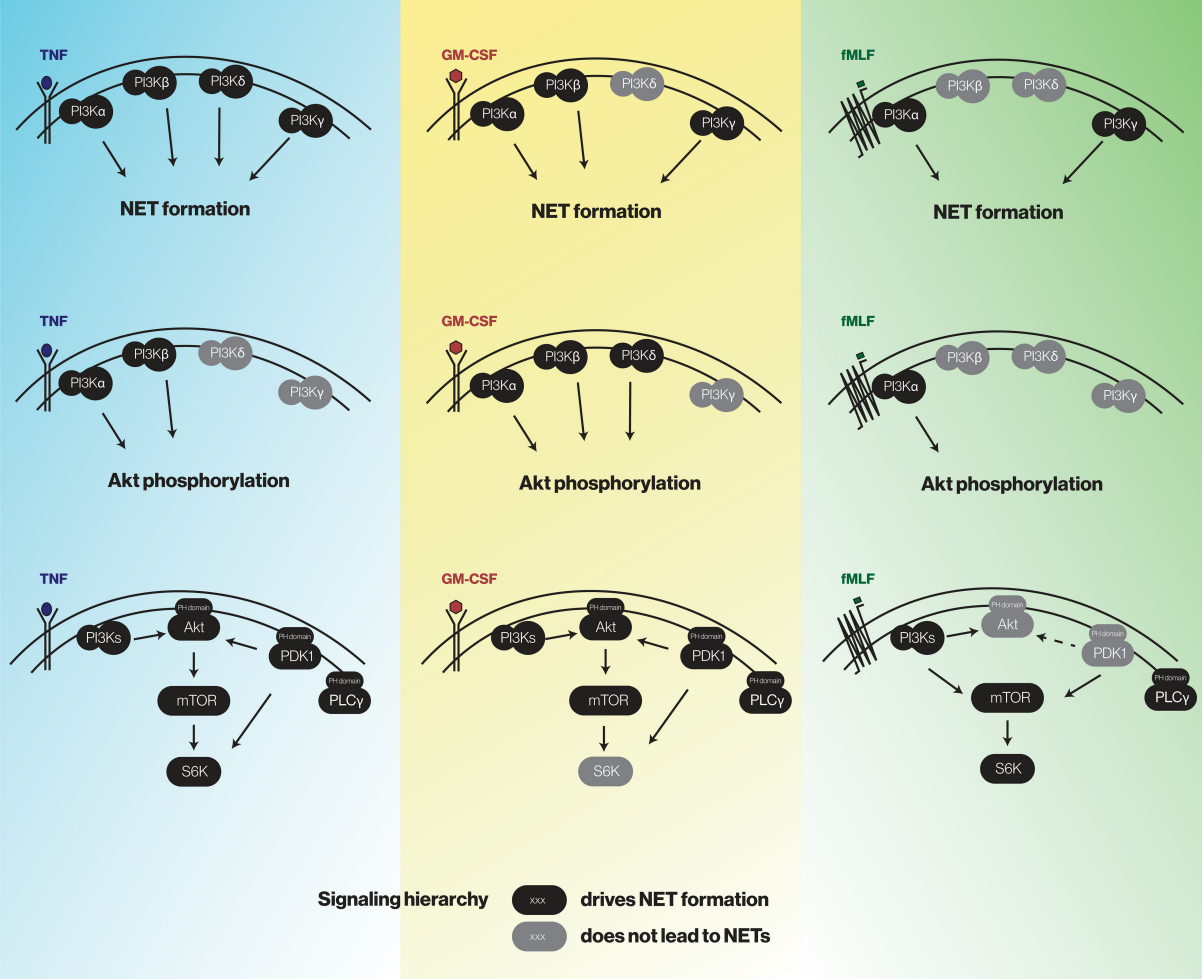
A summary of the findings of this study.

After having determined how discrete intermediates within the PI3K signaling pathway contribute to NET formation, we investigated whether their signaling hierarchy behaves similarly. To this end, neutrophils were pretreated with various pharmacological inhibitors; stimulated with either TNFα, GM-CSF, or fMLF; and immunoblot analyses were carried out for P-Akt, P-mTOR, and P-S6K. As shown in **Figure 7**, PI3K lies upstream of all the kinases analyzed regardless of the stimulus, except in the case of GM-CSF-elicited P-mTOR. Similarly, PDK1 largely controls all the kinases investigated in TNF- and GM-CSF-stimulated neutrophils, whereas in fMLF-activated cells, it moderately (though significantly) affects the phosphorylation of Akt and mTOR, but not that of S6K (**Figure 7**). By contrast, Akt inhibition exerts little or no effect on mTOR or P-S6K except in GM-CSF-treated cells where it strongly affected P-S6K levels (**Figure 7**). Finally, mTOR was found to control S6K phosphorylation in response to all stimuli tested (**Figure 7**). The findings of this paper are summarized in **Figure 8**.

## Discussion

Nearly two decades after the discovery of NETs, great leaps have been made in understanding the signaling cascades controlling this response. In particular, numerous reports have established that the PI3K pathway is a crucial driver of NET production (10–14). However, the intricacies of the signaling by, or related to, PI3K have remained heretoforth largely unexplored. In this report, we undertook a systematic study of how the PI3K cascade affects NET formation. We now demonstrate that discrete PI3K isoforms are mobilized in a stimulus-dependent manner to drive NET generation. We further reveal the stimulus-dependent involvement of distinct PI3K signaling partners in this emblematic neutrophil response. And in agreement with our previous finding that PI3K affects NET generation belatedly (13, 14), we show that all PI3K-related signaling molecules contributing to NET induction likewise act long after stimulation.

Our results demonstrate that there exist conserved, as well as stimulus-specific PI3K isoforms driving NET formation. Across various classes of neutrophil stimuli, PI3Kα and PI3Kγ isoforms clearly contribute to NET induction. In growth factor- or chemoattractant-stimulated cells, PI3Kβ was also involved but PI3Kδ was not. Conversely, in TNF-stimulated neutrophils, PI3Kδ was indispensable and PI3Kβ might also contribute to NET generation – in keeping with previous studies (from our laboratory and others) showing that other TNF-induced neutrophil responses rely on PI3Kβ and PI3Kδ (16–18). Thus, both class IA and class IB PI3K enzymes contribute to NET formation, albeit with stimulus-dependent differences. This is consistent with previous reports showing that in human neutrophils, there can be redundancy amongst class IA and IB PI3Ks in GM-CSF-stimulated cells (35), and that class IA and IB PI3Ks are sequentially mobilized in fMLF-activated cells (36). Likewise, it was reported that PI3Kδ and/or PI3Kγ mediates NET formation elicited by parasites or fungi (21, 22, 37). Thus, our conclusion that both class IA and class IB PI3Ks contribute to NET formation extends beyond the various soluble stimuli studied herein, and might represent a general feature of NET formation. Our data also shows that Akt, the prototypical kinase downstream of PI3K that is strongly phosphorylated in response to all stimuli tested herein, is activated by PI3Kα, PI3Kβ, and/or PI3Kδ; but not by PI3Kγ. It follows that in neutrophils, class IA PI3Ks influence NET formation (at least partially) by acting upon downstream Akt, whereas class IB PI3Ks do so independently of Akt.

The stimulus-dependent mobilization of a discrete set of PI3K isoforms to induce NET formation was also reflected in a differential contribution of PI3K signaling partners to this neutrophil response. A conserved kinase that stood out across the various classes of stimuli which we tested, was mTOR. In this regard, there are conflicting reports about the actual role of this signaling intermediate, with some groups describing that mTOR inhibition decreases NET production while others reporting that mTOR has little effect on this response, or even augments it (23, 26, 27, 38–40). This is likely to result from the varied approaches utilized to assess NET production – with different incubation conditions, detection reagents, microscopic observation (or lack thereof), and the fact that nonspecific fluorescence is seldom taken into account. The latter consideration might also explain why in many such studies NET induction was modest, which can complicate interpretation. In this study, all of these parameters were controlled for; NET induction was robust; and all classes of stimuli were compared in exactly the same manner. As a result, we are confident in our finding, that mTOR is needed for NET generation, even though its inhibition never completely prevented the phenomenon. Our signaling studies showed that mTOR is controlled (to varying degrees) by PI3K, PDK1, and Akt; a notable exception was GM-CSF-stimulated cells, in which mTOR was not under PI3K control. We further found that mTOR acts upstream of S6K, which also participates in NET formation in TNF-stimulated cells. Thus, GM-CSF-elicited NET generation involves both PI3K and mTOR, acting independently of each other; and while mTOR controls S6K activation under these conditions, S6K does not contribute to NET formation. Overall, mTOR is a conserved driver of NET formation, albeit with stimulus-dependent particularities. The underlying reason might have to do with the existence of mTOR splicing isoforms which are all sensitive to rapamycin inhibition (41); whether this is the case in neutrophils is currently unknown.

Other PI3K signaling partners involved in NET formation were Akt (which was conserved among the various stimuli used) and PDK1. In the case of Akt, our data is in keeping with similar findings made in neutrophils exposed to nonphysiological stimuli (PMA, calcium ionophore) or *Leishmania* parasites (23–25). Our signaling studies also revealed that in response to TNFα or GM-CSF, most of the signaling intermediates within the PI3K cascade, including Akt, were under dual control by both PI3K and PDK1. Conversely, we found that PDK1 can be dispensable for NET formation as the kinase was not involved in fMLF-elicited NET induction, despite a strong induction of Akt, mTOR, and S6K phosphorylation. Accordingly, we found that while activation of the latter kinases was firmly under the control of PI3K in fMLF-treated cells, they were only modestly affected by PDK1 blockade. In the particular case of Akt, it is noteworthy that commercially available P-Akt S473 antibodies indiscriminately recognize the three known Akt isoforms (Akt1, Akt2, Akt3). Among those, at least Akt1 is expressed in human neutrophils (42) and it is likely that other two isoforms are also present, since mouse neutrophils as well as human neutrophil-like HL-60 cells express all three isoforms (43–45). This raises the possibility that Akt isoforms whose activation involve PDK1, impact NET formation more profoundly – as observed in TNF- or GM-CSF-treated cells, whereas in fMLF-activated neutrophils, Akt participation in NET generation was modest. Alternatively, various Akt isoforms might simply be more strongly phosphorylated when PDK1 is mobilized by a given stimulus. Further studies will be needed to address this question. Unexpectedly, we found that NET generation induced by all stimuli tested depends on PLCγ2. Before the present study, PLCγ2 had been implicated in NET formation in response to CXCL8 but not PMA or ionomycin (23). The reliance on PLCγ2 could therefore represent yet another difference between physiological neutrophil agonists and pharmaceutical inducers, as already observed in the case of ROS and elastase involvement (13, 14, 29). In any instance, it emerges that fMLF-elicited, PI3K-dependent NET formation is mostly or completely unaffected by Akt and PDK1, but relies on conserved molecules (mTOR, S6K) as well as PLCγ2. By comparison, TNFα and GM-CSF relied on PDK1, Akt, mTOR, and PLCγ2 to induce NETs.

In conclusion, this study significantly advances our understanding of how the all-important PI3K pathway controls NET formation, by identifying both conserved and stimulus-dependent PI3K isoforms and downstream signaling molecules contributing to this emblematic neutrophil response. Because some of these PI3K partners (notably Akt) can translocate to the nucleus in other cell types (46, 47), it will prove interesting to explore the possibility that PI3K signaling might occur in different intracellular locations in neutrophils. In a broader context, our findings are of particular relevance given the role of NETs in health and disease, as they reveal therapeutic targets that can be exploited in both the near and more distant future. In this regard, drugs targeting PI3K-related enzymes are already used in the clinic, and more are being developed (48). For instance, the PI3Kα inhibitor Alpelisib is FDA-approved to treat metastatic breast cancer as well as head and neck cancer. Likewise, rapamycin (a mTOR inhibitor, also known as Sirelimus) is used to treat certain malignant tumors, and Akt inhibitors (such as MK2206) are undergoing clinical trials for cancer therapy. In view of our finding that PI3Kα, Akt and mTOR are conserved NET drivers across all stimuli tested, and given the role of NETs in awakening dormant cancer cells and promoting tumor invasion and distant metastases (49–53), it is conceivable that in cancer patients, inhibitors of PI3Kα, Akt and mTOR exert at least a part of their actions by interfering with NET formation. This also suggests that the same inhibitors could be used to treat other NET-driven diseases. Likewise, our work indicates that PI3Kγ and PLCγ2 inhibitors could act in similar fashion and therefore also represent promising therapeutic tools.

## Data availability statement

The original contributions presented in the study are included in the article/**Supplementary Material**. Further inquiries can be directed to the corresponding author.

## Ethics statement

The studies involving human participants were reviewed and approved by Comité d’éthique de la recherche du CIUSSS de l’Estrie – CHUS; Centre de recherche du CHUS (CRCHUS), Sherbrooke, Qc Canada J1H5N4. The patients/participants provided their written informed consent to participate in this study.

## Author contributions

VCO carried out most of the experiments, provided conceptual input, compiled all the data, and wrote the first draft; OT initiated the project and provided conceptual input; PMcD designed the research, mentored the other authors, and wrote the final version of the paper. All authors contributed to the article and approved the submitted version.
