# Supplementary material for: Phosphoinositol 3-kinase-driven NET formation involves different isoforms and signaling partners depending on the stimulus
Source: Front Immunol. 2023 Jan 24;14:1042686. doi: 10.3389/fimmu.2023.1042686 (PMC9904237; doi:10.3389/fimmu.2023.1042686)
Supplement: Supplementary file 1 [file DataSheet_1.pdf]

Supplementary Material

Phosphoinositol 3-kinase-driven NET formation involves different isoforms and signaling partners depending on the stimulus

Vanessa de Carvalho Oliveira, Olga Tatsiy, and Patrick P. McDonald*

*** Correspondence:** patrick.mcdonald@USherbrooke.ca

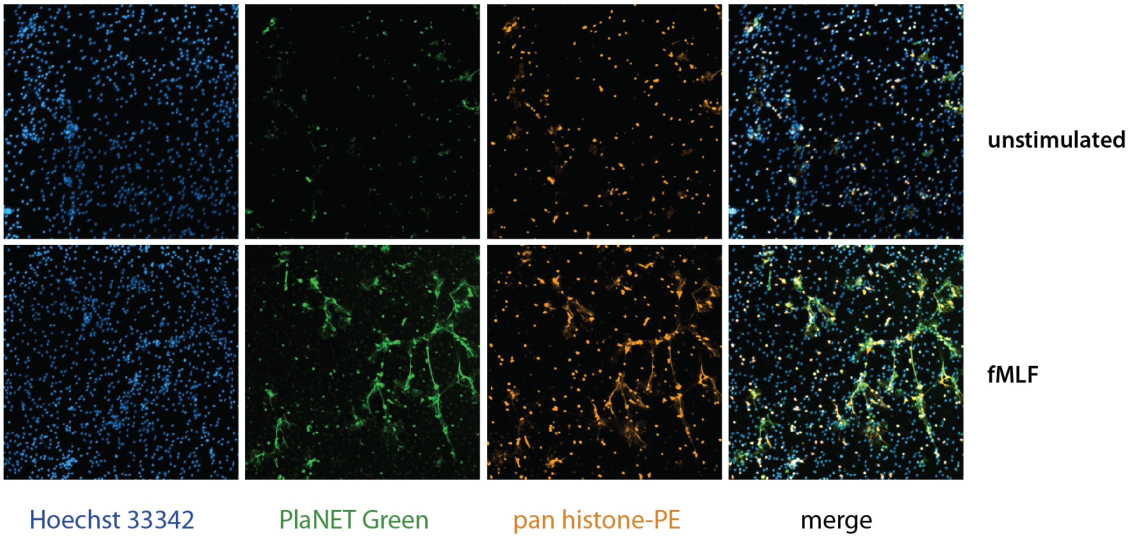

Figure S1. NET staining using PlaNET Green and another NET constituent. Neutrophils were cultured at 37°C on poly-L-lysine-coated coverslips for 4 h in the presence or absence of 100 nM fMLF. The cells were then fixed in the presence of the nuclear dye, Hoechst 33442, prior to staining with PlaNET Green and a pan-histone Ab coupled to a PE fluorophore. Cells were then fixed again and analyzed by epifluorescence microscopy (10X magnification).

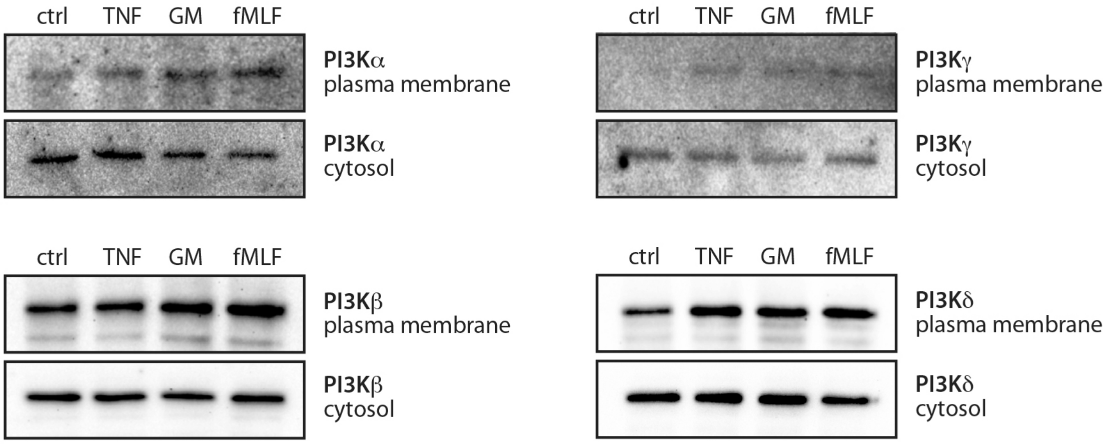

Figure S2. Membrane mobilization of PI3K isoforms in human neutrophils. Cells were stimulated or not ("ctrl") for 10 min at 37°C with 100 U/ml TNFα, 1 nM GM-CSF, or 100 nM fMLF. Reactions were stopped, cells were disrupted by nitrogen cavitation, and subcellular fractions were prepared as previously described (28). Cytosolic and plasma membrane fractions volumes were standardized based on total protein content for each series of fractions. Samples were processed for immunoblot analysis of the depicted PI3K isoforms; about 7 x 10^7 neutrophil equivalents were loaded on the gels for membrane fractions, and about 2 x 10^5 neutrophil equivalents for cytosolic fractions.

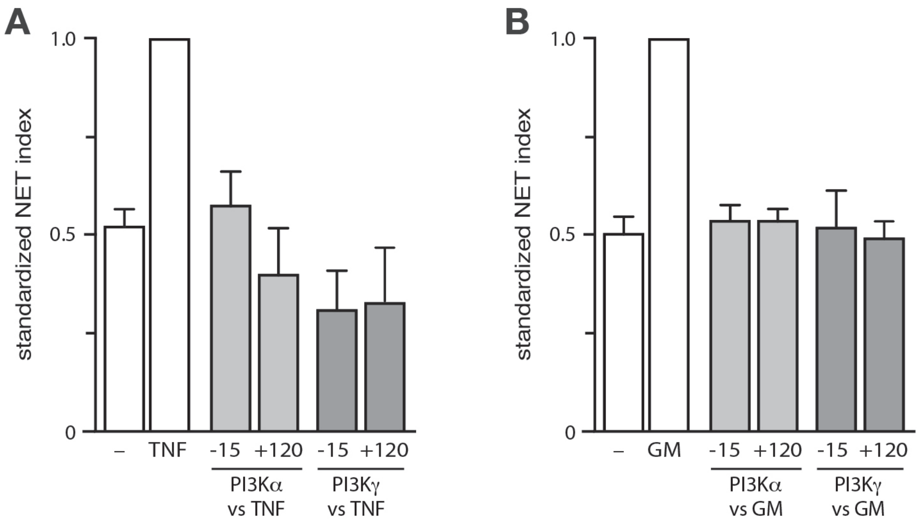

Figure S3. Belated inhibitory effect of PI3K isoforms on NET production. Neutrophils cultured on poly-L-lysine-coated coverslips were treated, either before (15 min, 37°C) or into (+120 min) a 4-h stimulation with 100 U/ml TNFα or 1 nM GM-CSF, with the following PI3K isoform-selective inhibitors: 1 µM PI3Kα inhibitor IV or 1 µM AS64002 (PI3Kγ inhibitor). NET formation was assessed using PlaNET Green as described in Methods. A representative experiment is shown.

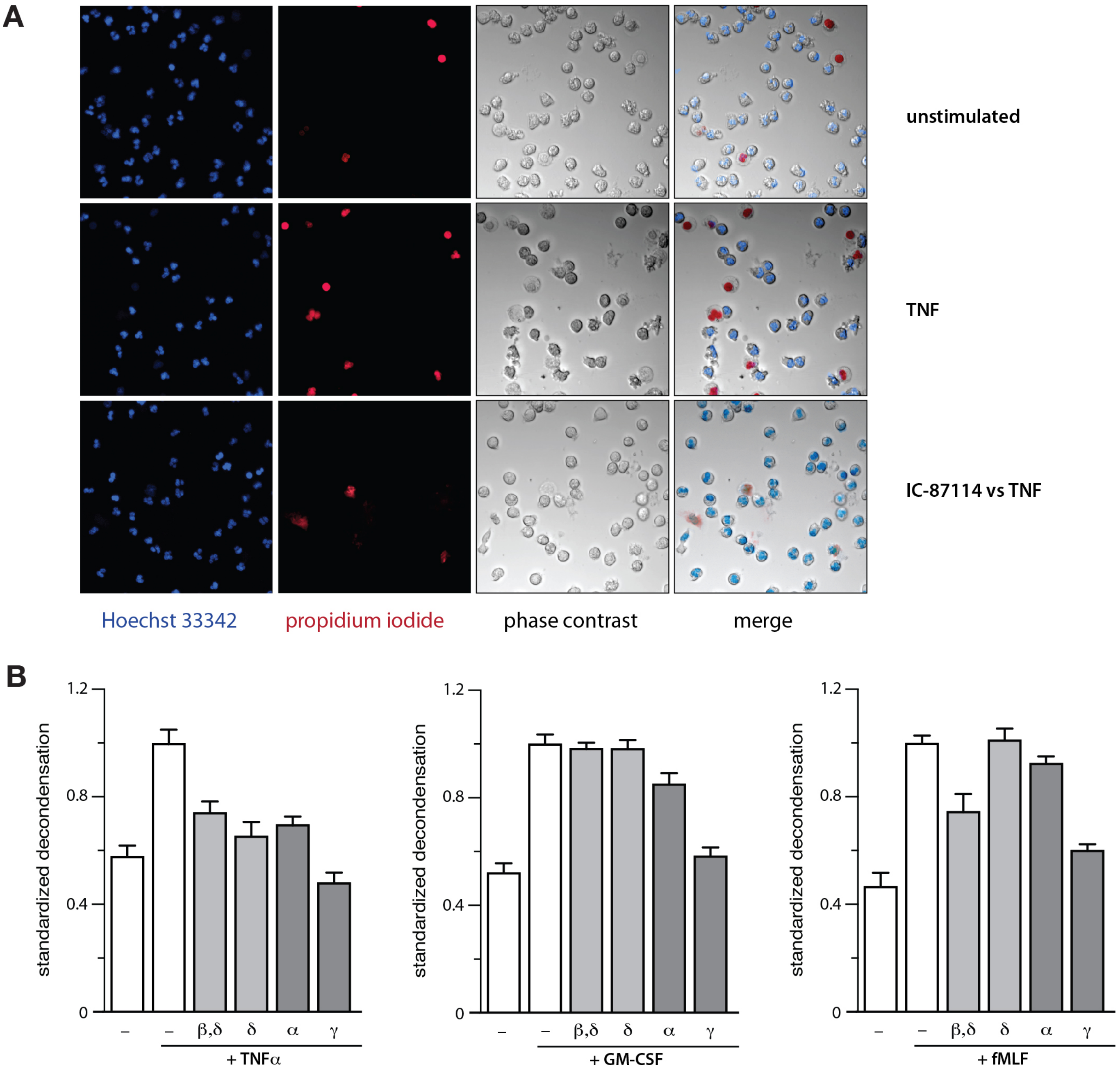

Figure S4. Effect of PI3K isoform inhibitors on chromatin decondensation.
Neutrophils cultured at 37°C on poly-L-lysine-coated coverslips were pre-treated (15 min) with the following PI3K isoform-selective inhibitors or their diluent (0.1% DMSO): 10 µM LY294002 (a general PI3K inhibitor, "LY"); 1 µM TGX-221 (β, δ); 1 µM IC87114 (δ); 1 µM PI3Kα inhibitor IV (α); 1 µM AS64002 (γ). The cells were then incubated at 37°C for 2.5 h in the absence or presence of 100 U/ml TNFα, 1 nM GM-CSF, or 100 nM fMLF. At this point, the following nuclear dyes were added: Hoechst 33342 (cell-permeable, to stain all nuclei) and propidium iodide (cell-impermeable, to stain cells whose membrane had ruptured). The cells were then further incubated at 37°C for another 30 min (3.5 h total stimulation time) before confocal microscope analysis. **(A)** Representative micrographs are shown (40X magnification) for TNF-treated cells. **(B)** Quantitative compilation of data from the same experiment, for which the % cells with nuclei showing decondensed chromatin was standardized to the value obtained for stimulated cells. Mean ± s.e.m. of 6 replicates for each experimental condition.

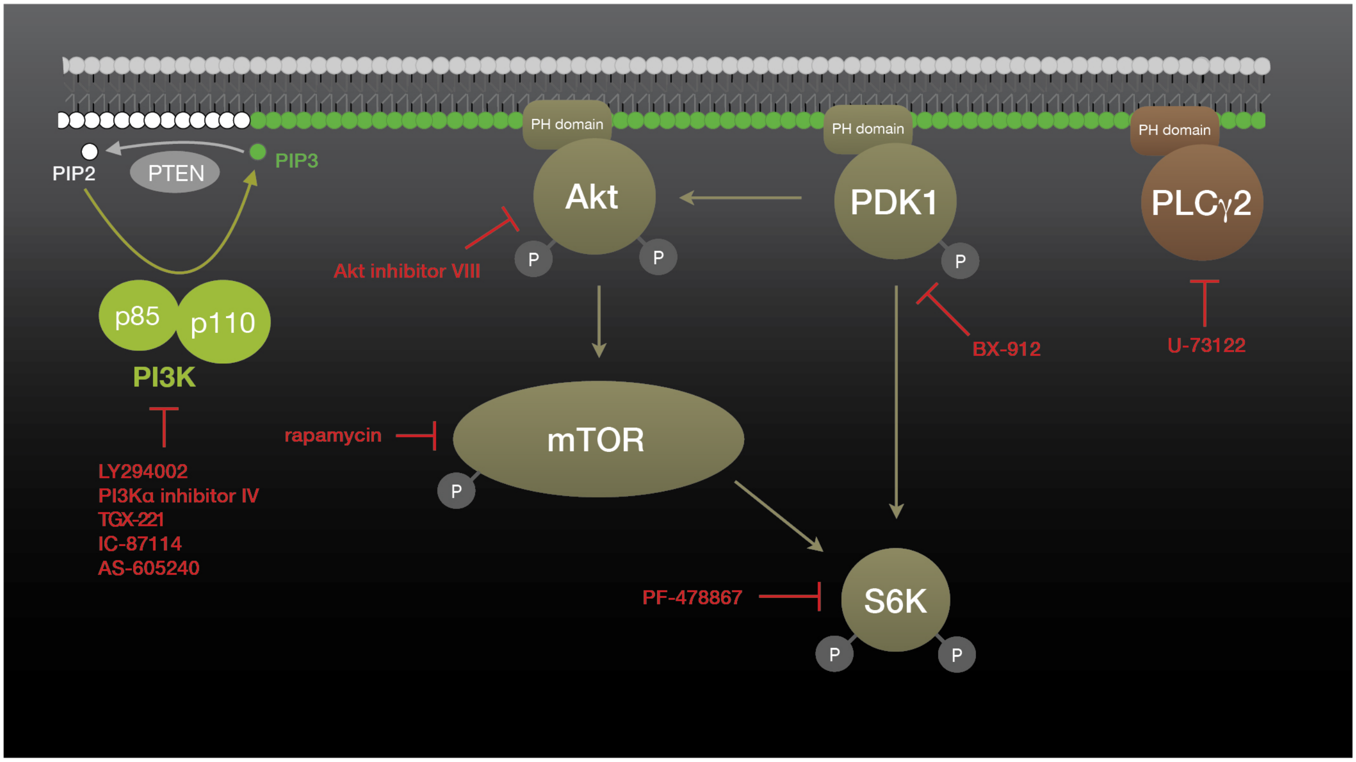

Figure S5. The various kinase inhibitors used in this study.

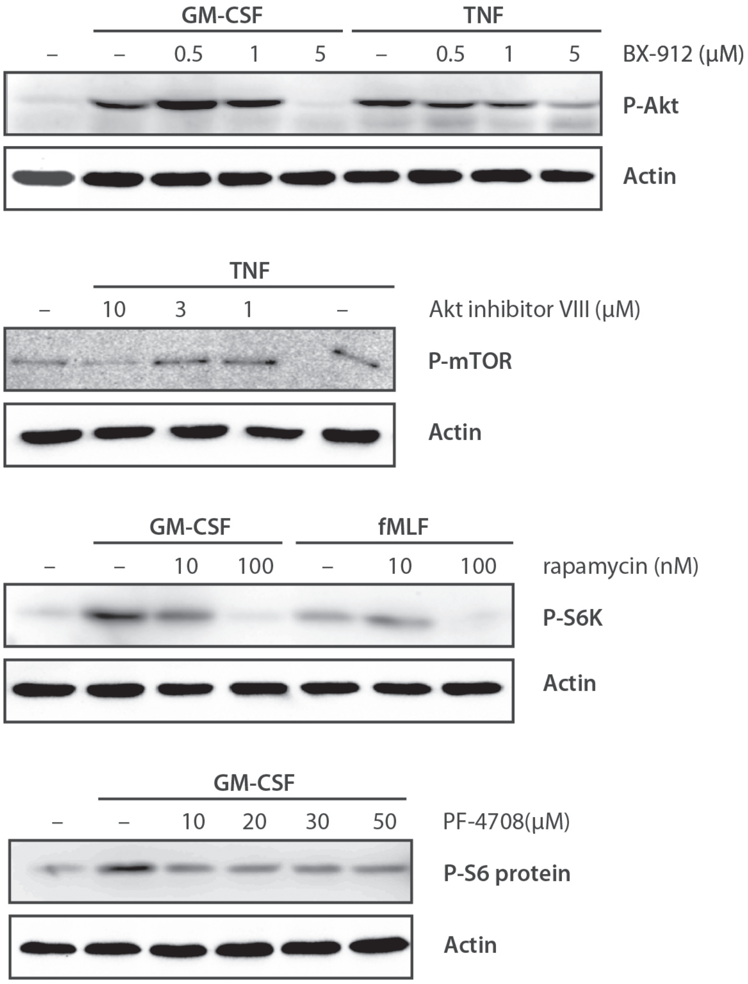

Figure S6. Dose responses to inhibitors of various PI3K-related signaling intermediates in human neutrophils. Cells were pre-treated (15 min, 37°C) with the following inhibitors or their diluent (0.1% DMSO) at the indicated concentrations: BX-912 (PDK1 inhibitor); Akt inhibitor VIII (Akt inhibitor); rapamycin (mTOR inhibitor); or PF-4708671 (S6K inhibitor). Neutrophils were then stimulated or not (15 min, 37°C) with 100 U/ml TNFα, 1 nM GM-CSF, or 100 nM fMLF. The samples were processed for immunoblot analysis of P-Akt (S473), P-mTOR (S2448), P-S6K (T389), or P-S6 protein; as well as β-actin (loading control).

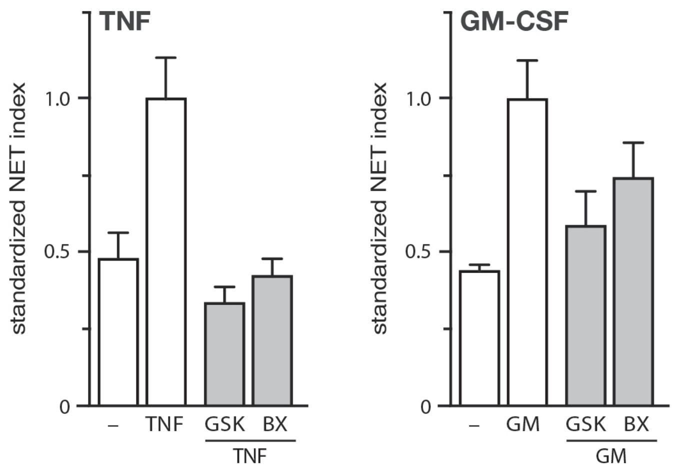

Figure S7. Effect of various PDK1 inhibitors towards NET formation.
Neutrophils cultured on poly-L-lysine-coated coverslips were pre-treated (15 min, 37°C) with 500 nM GSK 2334470, 500 nM BX-795, or their diluent (0.1% DMSO) prior to a 4-h stimulation with 100 U/ml TNFα or 1 nM GM-CSF. NET formation was assessed using PlaNET Green as described in Methods. Each of the depicted experiments is representative of two.

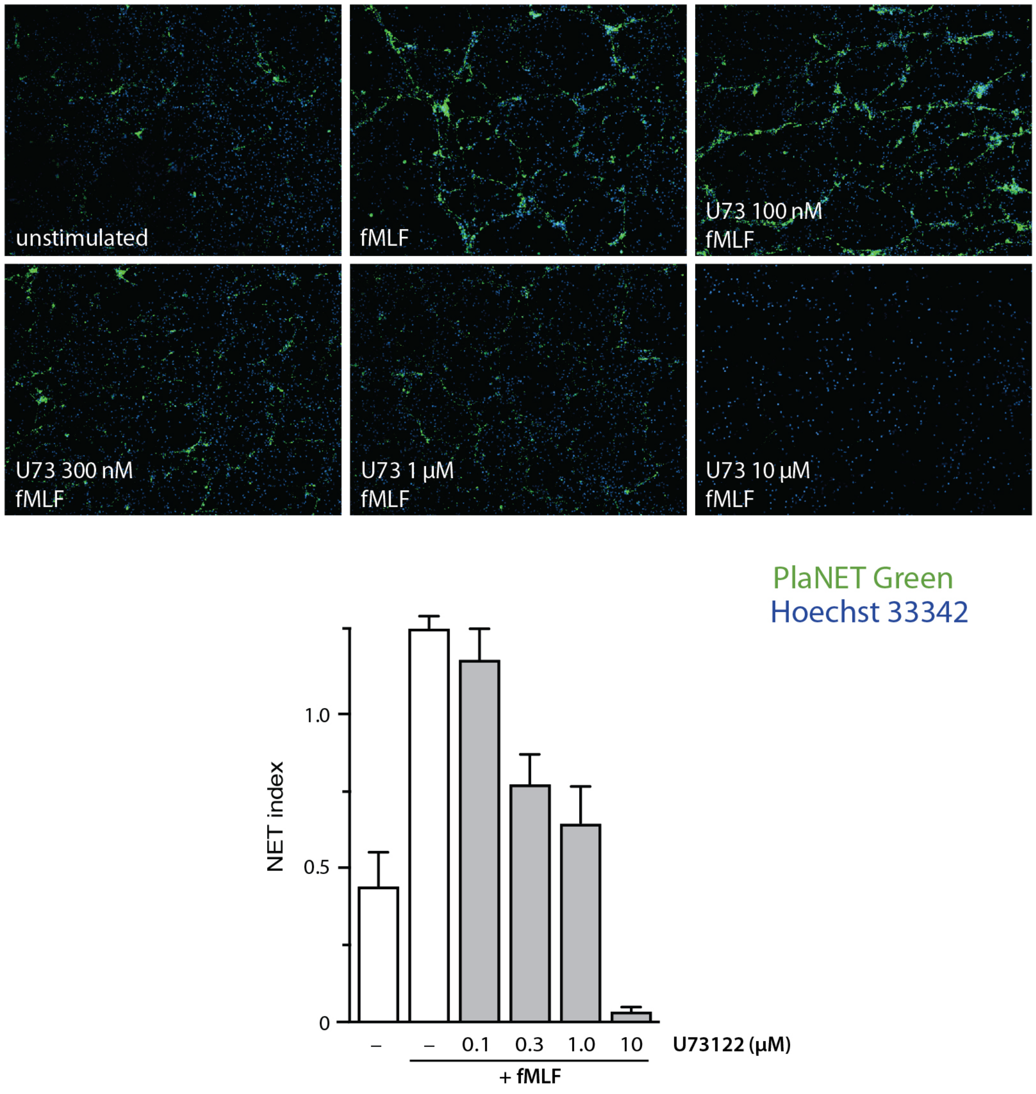

Figure S8. Dose response to U73122 pretreatment and its effect on NET induction. Neutrophils cultured on poly-L-lysine-coated coverslips were pre-treated (15 min, 37°C) with the indicated concentrations of U73122 (PLCγ2 inhibitor) or its diluent (0.1% DMF) prior to a 4-h stimulation with 100 nM fMLF. NET formation was assessed using PlaNET Green as described in Methods. The depicted experiment is representative of two.

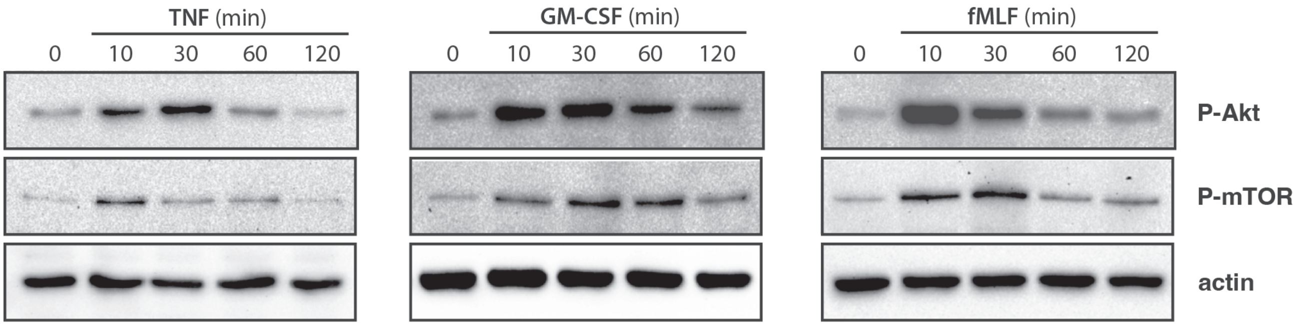

Figure S9. Time course of PI3K-related kinase phosphorylation in adherent neutrophils. Cells were cultured at 37°C on poly-L-lysine-coated coverslips for the indicated times in the presence of 100 U/ml TNFα, 1 nM GM-CSF, or 100 nM fMLF. Samples were then processed for immunoblot analysis of P-Akt (S473), P-mTOR (S2448), as well as β-actin (loading control). Each of the depicted experiments is representative of two.

Table S1. IC50 values for PI3K inhibitors towards individual PI3K isoforms. All concentrations are expressed in μM.

	PI3Kα	PI3Kβ	PI3Kδ	PI3Kγ	concentration used herein	references
LY294002	0.5	0.97	0.57	-	10	Chaussade *et al.* PMID: 17869522
TGX-221	5	0.007	0.1	3.5	1	Condliffe *et al.* PMID: 15878979
IC-87114	-	75	0.5	29	1	Sadhu *et al.* PMID: 12927784
PI3Kα inhibitor IV	0.002	0.016	-	0.66	1	Hayakawa *et al.* PMID: 16837202
AS-605240	0.06	0.27	0.3	0.008	1	Camps *et al.* PMID: 16127437
